# Transcriptional Pathways in cPGI_2_-Induced Adipocyte Progenitor Activation for Browning

**DOI:** 10.3389/fendo.2015.00129

**Published:** 2015-08-17

**Authors:** Irem Bayindir, Rohollah Babaeikelishomi, Silvia Kocanova, Isabel Sofia Sousa, Sarah Lerch, Olaf Hardt, Stefan Wild, Andreas Bosio, Kerstin Bystricky, Stephan Herzig, Alexandros Vegiopoulos

**Affiliations:** ^1^DKFZ Junior Group Metabolism and Stem Cell Plasticity, German Cancer Research Center, Heidelberg, Germany; ^2^University of Toulouse (UPS), Toulouse, France; ^3^Laboratoire de Biologie Moléculaire des Eucaryotes, Centre national de la recherche scientifique, Toulouse, France; ^4^Department of Life Sciences, University of Coimbra, Coimbra, Portugal; ^5^Miltenyi Biotec GmbH, Bergisch Gladbach, Germany; ^6^Helmholtz Center Munich, Institute for Diabetes and Cancer IDC, Neuherberg, Germany; ^7^Joint Heidelberg-IDC Translational Diabetes Program, Heidelberg University Hospital, Heidelberg, Germany

**Keywords:** beige/brite differentiation, adipocyte progenitors, prostacyclin, PGI_2_, adipocyte cell model, adipose tissue remodeling, nuclear localization

## Abstract

*De novo* formation of beige/brite adipocytes from progenitor cells contributes to the thermogenic adaptation of adipose tissue and holds great potential for the therapeutic remodeling of fat as a treatment for obesity. Despite the recent identification of several factors regulating browning of white fat, there is a lack of physiological cell models for the mechanistic investigation of progenitor-mediated beige/brite differentiation. We have previously revealed prostacyclin (PGI_2_) as one of the few known endogenous extracellular mediators promoting *de novo* beige/brite formation by relaying β-adrenergic stimulation to the progenitor level. Here, we present a cell model based on murine primary progenitor cells defined by markers previously shown to be relevant for *in vivo* browning, including a simplified isolation procedure. We demonstrate the specific and broad induction of thermogenic gene expression by PGI_2_ signaling in the absence of lineage conversion, and reveal the previously unidentified nuclear relocalization of the *Ucp1* gene locus in association with transcriptional activation. By profiling the time course of the progenitor response, we show that PGI_2_ signaling promoted progenitor cell activation through cell cycle and adhesion pathways prior to metabolic maturation toward an oxidative cell phenotype. Our results highlight the importance of core progenitor activation pathways for the recruitment of thermogenic cells and provide a resource for further mechanistic investigation.

## Introduction

The abundance and activation of thermogenic adipocytes are associated with improved metabolic health and protection from obesity, impaired glucose tolerance and dyslipidemia, at least as proven in diverse mouse models (1). Along with the discovery of functional thermogenic adipocytes in humans, this fact has potentiated research efforts toward understanding the biology of thermogenic adipocytes (2, 3). Beyond classical brown adipose tissue (BAT) depots, thermogenic adipocytes can be recruited and activated in other fat depots of rodents in the context of a tissue remodeling process from a lipid storing to an oxidative/thermogenic phenotype. The recruitment of these so-called beige or brite adipocytes occurs under conditions of prolonged cold exposure, β3-adrenoreceptor agonist treatment, and possibly physical exercise and environmental enrichment (2). The degree of recruitment has been shown to depend on the anatomical location of the fat depots as well as the genetic background.

The cellular origin of multilocular beige/brite adipocytes expressing uncoupling protein-1 (Ucp1) has not been fully determined. However, different mechanisms appear to occur in parallel (1, 2). On the one hand, multiple reports described the derivation of beige/brite adipocytes from unilocular “white-appearing” adipocytes, implying a metabolic conversion (4–6). On the other hand, a substantial proportion of beige/brite adipocytes were shown to be recruited through adipogenic differentiation of immature progenitor cells *in vivo* and in primary cultures (7–9).

The master signal driving thermogenic adipose tissue remodeling is provided by sympathetic nerves via norepinephrine (NE) and β-adrenergic signaling (1, 10). We have previously described cyclooxygenase (COX)-2-derived prostaglandins as some of the very few endogenous mediators reported to act on progenitor cells to promote beige/brite differentiation during β-adrenergic stimulation (8). We demonstrated that prostaglandin synthesis was acutely increased in β-adrenergically stimulated adipose tissue, and importantly, COX-2 function was required for browning of white adipose tissue, a finding confirmed in an independent report (11). Furthermore, we identified prostacyclin (PGI_2_) as a key prostaglandin downstream of COX-2. We could show that signaling induced by the stable analog carbaprostacyclin (cPGI_2_) promoted beige/brite differentiation in mouse and human primary progenitor cells from white fat (8). PGI_2_ can signal through the Ptgir G-protein-coupled receptor as well as through direct activation of all three members of the peroxisome proliferator-activated receptor (Ppar) family (12, 13). We could show that the full activation of the thermogenic program in progenitor cells as well as *in vivo* was dependent on signaling through both the Ptgir and Pparg receptors (8).

Despite the identification of a number of key regulatory nodes required for browning (1), we are far from understanding the signaling and transcriptional pathways regulating beige/brite differentiation downstream of extracellular mediators. This is partly due to the paucity of physiological cell models. Here, we describe a cell model for beige/brite differentiation based on adipogenic progenitors with defined surface markers and present a simplified method for their prospective isolation. Furthermore, we profile the cascade of progenitor cell responses to cPGI_2_ throughout differentiation and show that progenitor activation by cPGI_2_ via cell cycle and adhesion pathways precedes and synergizes with cPGI_2_-induced metabolic maturation of beige/brite adipocytes.

## Materials and Methods

### Mice

Female NMRI mice (Charles River WIGA GmbH, Sulzfeld, Germany) or C57BL/6N mice from bred in the internal facility were housed at ambient temperature with 12-h light–dark cycle on chow (Kliba Nafag #3437, Provimi Kliba, Kaiseraugst, Switzerland). Stromal-vascular fraction (SVF) FACS profiles were not significantly different and beige/brite differentiation capacity was comparable between the two strains across numerous-independent experiments (data not shown). The RNA expression profiling data were obtained from NMRI cells. Animal handling and experimentation were performed in accordance with the European Union directives and the German animal welfare act (Tierschutzgesetz) and approved by local authorities (Regierungspräsidium Karlsruhe).

### Adipose tissue digestion and SVF preparation

Posterior subcutaneous adipose tissue (gluteal + inguinal) or the brown part of the interscapular fat was dissected, minced with scissors, and digested with 0.1 w.u./ml purified collagenase (LS005273, Worthington Biochemical, Lakewood, NJ, USA) and 2.4 U/ml Neutral Protease (LS02104, Worthington Biochemical) in Hank’s balanced salt solution (HBSS, Sigma-Aldrich, Munich, Germany) containing 4 mM calcium chloride and 0.05 mg/ml DNase I (1284932001, Roche Diagnostics, Grenzach-Wyhlen, Germany) for 50 min at 37°C in a shaker. The suspensions were strained through a 300 μ mesh (4-1411, Neolab, Heidelberg, Germany). Floating mature adipocytes and SVF were separated by centrifugation at 145 × *g* for 10 min at 20°C. SVF cells were washed, and centrifuged at 300 × *g* for 5 min at 20°C.

### FACS analysis/sorting of SVF cells

Stromal-vascular fraction single cell suspensions in D-PBS (Life Technologies, Darmstadt, Germany) supplemented with 0.5% BSA and 1 mM EDTA (Sigma-Aldrich) were preincubated with FcBlock [anti-CD16/32 (93, eBioscience), Frankfurt, Germany] for 10 min on ice. Cells were then incubated with Anti-Ter119 MicroBeads (130-049-901, Miltenyi Biotec, Bergisch Gladbach, Germany) on ice for 15 min, to perform erythrocyte depletion by magnetic-activated cell sorting (MACS^®^) with an OctoMACS Separator according to the manufacturer’s instructions. The flow-through was collected and stained with CD45-FITC (30-F11, eBioscience), CD31-eFluor 450 (390, eBioscience), CD29-PerCP-eFluor 710 (HMb1-1, eBioscience), CD34-Alexa Fluor 647 (RAM34, BD Biosciences, Heidelberg, Germany), Sca-1-Alexa Fluor 700 (D7, eBioscience), and CD140a(Pdgfrα)-biotin (APA5, eBioscience) for 30 min on ice, followed by staining with streptavidin-PE-Cy7 (eBioscience). After antibody staining, samples were washed and sorted with a BD FACS Aria (BD Biosciences). Unstained cells as well as FMO stainings were used as negative controls. Single-stained controls were used for compensation. Data were analyzed using FlowJo software (FlowJo, Ashland, OR, USA). Sorted cells were centrifuged at 300 × *g* for 5 min for further processing.

### MACS^®^ cell isolation

Stromal-vascular fraction single cell suspensions in D-PBS (Life Technologies) supplemented with 0.5% BSA and 1 mM EDTA (Sigma-Aldrich) were preincubated with FcBlock (anti-CD16/32 (93, eBioscience, Frankfurt, Germany) for 10 min on ice. Cells were then stained with biotin-conjugated Ter119 (TER-119), CD31 (390), and CD45 (30-F11) antibodies (eBioscience) for 30 min on ice, washed and incubated with Streptavidin MicroBeads (130-048-102, Miltenyi Biotec) for magnetic separation with an OctoMACS Separator according to the manufacturer’s instructions. The flow-through (Lin^−^ cells) was stained with Sca1-PE-Cy7 (D7, eBioscience) for 30 min on ice, washed and incubated with Anti-Cy7 MicroBeads (130-091-652, Milenyi Biotec) for magnetic separation according to the manufacturer’s instructions. The flow-through (Lin^−^Sca-1^+^ cells) was centrifuged at 300 × *g* for 5 min for further processing. Flow cytometry for the assessment of recovery/purity was performed on a FACScalibur (BD Biosciences). For the isolation of Lin^−^Sca^+^ cells without post-isolation flow cytometric analysis, Anti-Sca-1 MicroBeads (130-106-641) were used following the isolation of Lin^−^ cells or alternatively, the Adipose Tissue Progenitor Isolation Kit (130-106-639) on SVF isolated using the Adipose Tissue Dissociation Kit (130-105-808) in combination with the gentleMACS Octo Dissociator (130-096-427) (all Miltenyi Biotec).

### Cell culture and adipogenic differentiation

Media and supplements were purchased from Life Technologies (Darmstadt, DE) unless stated otherwise. Sorted (FACS or MACS) cells were plated out on BIOCOAT Laminin-coated plates (BD Biosciences) and maintained in DMEM plus 10% FCS, 1% penicillin/streptomycin, and 10 ng/ml murine bFGF (R&D Systems, Wiesbaden, DE) without passaging. Adipogenic differentiation of confluent cells was performed in the presence of 1 μM cPGI_2_ (BIOZOL, Eching, DE) throughout the course of differentiation (unless otherwise indicated) or corresponding concentration of ethanol as control (“white” cells). Media ± cPGI_2_ were replaced daily with fresh. Differentiation was induced with medium consisting of DMEM, 10% FCS, 1% penicillin/streptomycin, 1 μg/ml insulin, 500 nM dexamethasone, 3 nM triiodothyronine (T3) (Sigma-Aldrich) for 2 days. Subsequently, cells were cultured in differentiation medium with 5% FCS lacking dexamethasone for 5 days, and DMEM, 5% FCS, 1% penicillin/streptomycin for 1 day. Wherever indicated, 3 h before harvest of cultures, cells received fresh medium with and without 0.5 μM NE (Sigma-Aldrich) but omitting cPGI_2_.

### RNA isolation and qRT-PCR analysis

Cell lysis was performed in QIAZOL (QIAGEN, Hilden, Germany), and RNA was prepared using the RNeasy micro kit (QIAGEN) including DNase treatment. Reverse transcription was performed with 0.1–1 μg total RNA and oligo(dT) primers using Superscript II (Life Technologies). Quantitative PCR was performed with the TaqMan Universal Master Mix II and gene-specific Taqman probes on a StepOnePlus machine (Life Technologies, Darmstadt, DE). Relative mRNA expression levels were calculated with the ΔΔCt method and TATA-binding protein (*Tbp*) as a reference.

### Microarray expression profiling

Preparation of biotin-labeled cRNA samples from 500 ng total RNA, and hybridization on Illumina Mouse Sentrix-6 BeadChip arrays (Illumina, San Diego, CA, USA) were performed according to the manufacturer’s instructions. Scanning was performed on a Beadstation array scanner (Illumina). The raw microarray data are available in the ArrayExpress database^1^ under accession number E-MTAB-3693. Beads with a value >20 (bead level) were selected, and outliers with values >2.5 median absolute deviation (MAD) were removed. All remaining data points were used for the calculation of the mean average signal for a given probe. Intensity values were normalized by quantile normalization using Chipster Software (CSC, Espoo, Finland). Probe annotation was according to MouseWG-6_V2_0_R1_11278593_A. Principal component analysis was performed with TM4 MeV software on log2-transformed values using median centering (14). Two-group significance tests as indicated were performed in Chipster without pre-filtering of probes on log2-transformed values (empirical Bayes with Bonferroni-Holm *p*-value adjustment). Gene set enrichment analysis [GSEA (15)] was performed on the complete probe dataset based on the MouseWG-6_V1_1_R4_11234304_A annotation. The following gene set collections from the Molecular Signatures Database (MSigDB)^2^ were used: The c2.cp.kegg.v3.0.symbols.gmt gene set collection, derived from http://www.genome.jp/kegg/pathway.html, and the c3.tft.v3.0.symbols.gmt gene set collection, in with gene sets contain genes that share a transcription factor binding site defined in the TRANSFAC database^3^. The following key parameters were applied: permutation type = phenotype (1000×), enrichment statistic = weighted, metric for ranking genes = Signal2Noise, normalization mode = meandiv. Gene Sets were ranked by the false discovery rate.

### DNA–fluorescence *In situ* hybridization

Glass cover slips (HECH1001/12, Karl Hecht, Sondheim, Germany) were coated with 4 μg/cm^2^ laminin (Santa Cruz Biotechnology, Heidelberg, Germany) for 1 h at 37°C and washed with DMEM. Freshly isolated progenitor cells (see above) were plated on glass cover slips in growth and differentiation media, as described above. Cells were fixed at the indicated time points with fresh 4% paraformaldehyde. DNA–fluorescence *in situ* hybridization (FISH) experiments were performed, as previously described (16). DNA from BAC clones MSG01-182C14 for *Ucp1* and RP24-238M20 for *Pum1* purchased from DNA Bank, RIKEN and BACPAC C.H.O.R.I. Center, (USA), respectively, was directly labeled using nick translation (BioPrime DNA Labeling System, Life Technologies, Saint Aubin, France) by incorporation of fluorochrome-conjugated nucleotidesChromaTide^®^ AlexaFluor^®^ 488-5-dUTP (Life Technologies) for *Ucp1* and Atto647N-dUTP-NT (Jena Biosciences, Jena, Germany) for *Pum1*. One hundred nanogram of each labeled DNA probe together with 7 μg Cot-1 mouse DNA and 5 μg sonicated salmon sperm DNA were used per coverslip. Cells were examined by Nikon Ti-E/B epifluorescence microscope, equipped with a HG Intensilight^®^ illumination source, a CCD Orca R2 camera (Hamamatsu^®^) and imaged through an NIKON oil-immersion objective 60× (Plan APO 1.4). The devices were controlled by NIS-elements^®^ 3.2. Three-dimensional images were captured at 200 nm intervals in the *z*-axis, using an objective fitted with a piezo nano Z100 Ti. Analysis of nuclear position of the detected fluorescent signals was performed using NEMO software (17). The radial localizations of loci were then calculated in Microsoft Excel. Three shells of equal area eroded from the center (shell 1) to the periphery (shell 3) of the nucleus were used. Images from 30 to 50 nuclei were analyzed in each experiment. Finally, the images were processed using Adobe Photoshop.

### EdU incorporation analysis

Cells were incubated with 10 μM 5-ethynyl-2-deoxyuridine (EdU) in their normal medium for 1 h, trypsinized and washed. Fixation (4% paraformaldehyde, 15 min), permeabilization and the Click-it reaction for AlexaFluor647 labeling were performed using the Click-iT^®^ Plus EdU Flow Cytometry Assay Kit (Life Technologies, Darmstadt, Germany). Cells were treated with FxCycle™PI/RNase Staining Solution (Life Technologies) and analyzed on a FACSCalibur (BD Biosciences, Heidelberg, Germany).

### Statistical analysis

Plots depict means and SEM unless otherwise indicated. The corresponding test and significance level are indicated in the figure legends. Gene expression data were tested in the log-scale for approximation of normality. Two-way ANOVA was applied with Bonferroni *post hoc* pairwise test. One-way ANOVA was applied with Tukey *post hoc* pairwise test. Two-sided *t*-test was applied for two-group experiments. The statistical significance of differences in nuclear radial localization (FISH) was assessed using the chi-square (χ^2^) test to examine the null hypothesis that the foci exhibit the same radial distribution in both treatments (cPGI_2_ vs. Control). A *p*-value ≤0.05 was considered statistically significant.

## Results

### cPGI_2_ broadly and specifically induces the thermogenic gene expression program in Lin^−^CD29^+^CD34^+^Sca-1^+^Pdgfra^+^ progenitors without lineage conversion

We have previously demonstrated that beige/brite differentiation can be efficiently induced by cPGI_2_, a stable analog of PGI_2_, in Lin(Ter119/CD31/CD45)^−^CD29^+^CD34^+^Sca-1^+^ cells isolated by FACS from subcutaneous tissue (8). This population has been shown to contain all adipogenic cells in the SVF (18). Genetic lineage tracing *in vivo* has revealed that expression of Platelet-derived growth factor receptor a (Pdgfra) marks progenitors with both beige/brite and white adipogenic potential (7, 19). Importantly, it is the only marker proven so far by genetic lineage tracing to be broadly expressed in beige/brite progenitors *in vivo*. In accordance with Berry et al., we could confirm that the majority of the Lin^−^CD29^+^CD34^+^Sca-1^+^ population (>90%) was positive for Pdgfra expression (Figures S1A–C in Supplementary Material) (19), implying that it is likely to include most immature beige/brite progenitor cells.

**Figure 1:**
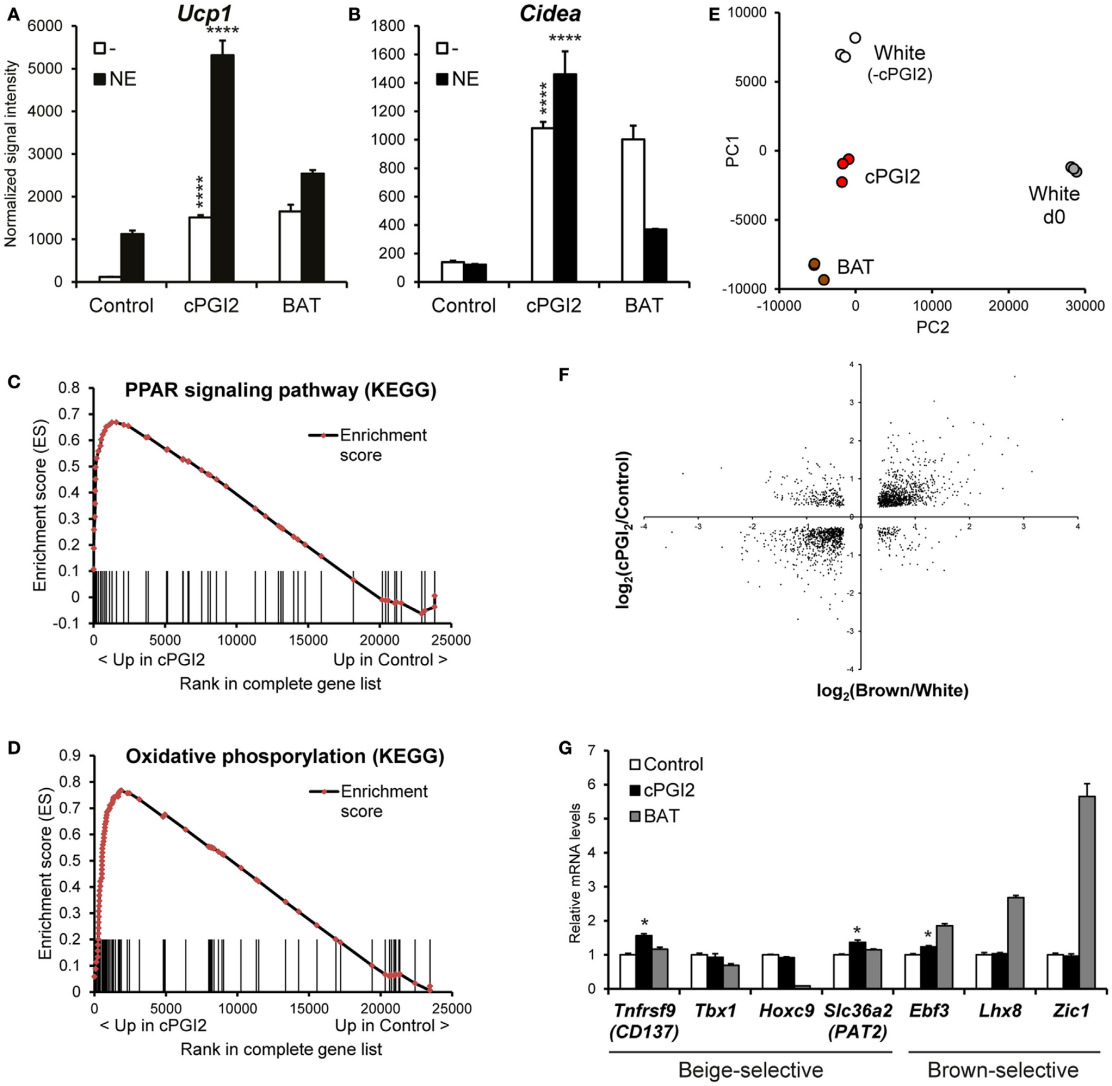
**cPGI_2_ specifically induces a broad thermogenic gene expression program in adipocyte progenitors without lineage conversion**. Lin^−^CD29^+^CD34^+^Sca-1^+^ cells from posterior subcutaneous fat were cultured in adipogenic media ± cPGI_2_ for 8 days. Lin^−^CD29^+^CD34^+^Sca-1^+^ cells from interscapular brown fat (BAT) were cultured in adipogenic media for 8 days and used as a reference. Three hours before harvest, cells were cultured ± norepinephrine (NE). RNA expression profiling was performed with Illumina beadchip arrays (*n* = 3). **(A,B)** Normalized signal intensities for the indicated genes/probes are shown (asterisks indicate Bonferroni cPGI_2_ vs. Control ****p < 0.0001, *p < 0.05, *n* = 3). **(C,D)** Enrichment plots of the OxPhos **(C)** and PPAR **(D)** gene sets obtained by GSEA (cPGI2 vs. Control) with the KEGG pathway gene set collection (FDR *q* ≤ 0.0001, see Table 1). Vertical bars represent the individual genes of the gene set/pathway ranked according to their regulation by cPGI_2_ (based on signal-to-noise ratio, see Materials and Methods). *X*-axis values represent the rank within the complete ranked gene list (transcriptome). The enrichment score (ES) reflects the degree to which a gene set is overrepresented at the top or bottom of the complete ranked gene list. **(E)** Principal component analysis was performed on RNA expression profiles from day 8 differentiated cells as indicated (White, cPGI2, BAT) including undifferentiated subcutaneous Lin^−^CD29^+^CD34^+^Sca-1^+^ cells (White d0). The sample coordinates for principal component (PC) 1 and 2 are shown. PC1 and PC2 captured 80% of the overall variability. **(F)** 1793 genes were selected with significant differential expression (*p* < 0.05) in both the cPGI_2_ vs. Control and the BAT vs. “white” (equivalent to Control, i.e., minus cPGI_2_) comparisons. The log2-ratios of the corresponding expression levels in the two comparisons were plotted. **(G)** Normalized signal intensities of the indicated genes are plotted (relative to Control). (* indicates Tukey cPGI_2_ vs. Control *p* < 0.01, *n* = 3).

In order to obtain a global picture of the differentiation phenotype induced by cPGI_2_ in progenitor cells, we performed time course expression profiling of Lin^−^CD29^+^CD34^+^Sca-1^+^ cells stimulated with cPGI_2_ under adipogenic conditions. As shown previously, cPGI_2_ robustly induced the thermogenic/brown adipocyte marker genes *Ucp1* and *Cidea* after 8 days of differentiation (Figures 1A,B) (8). *Ucp1* expression could be super-activated by NE, demonstrating the responsiveness of cPGI_2_-treated cells to this thermogenic inducer. Notably, expression levels of *Ucp1* and *Cidea* were comparable to adipocytes differentiated from Lin^−^CD29^+^CD34^+^Sca-1^+^ cells from interscapular BAT (Figures 1A,B). cPGI_2_ has been proposed to promote adipogenic differentiation (20). However, in our primary cell model, most adipogenic marker genes include Adiponectin (*Adipoq*) and Resistin (*Retn*) were not or only modestly and inconsistently induced by cPGI_2_ (Figures S2A,B in Supplementary Material, and data not shown).

We next performed GSEA (15) to examine the biological pathways induced in cPGI_2_-treated cells at 8 days of differentiation in an unbiased fashion. Figures 1C,D illustrate the enrichment of genes involved in oxidative phosphorylation and the PPAR signaling pathway, respectively, in the fraction of genes up-regulated by cPGI_2_. The oxidative phosphorylation gene set contains all the subunit genes of the respiratory chain complexes, and their upregulation by cPGI_2_ is consistent with mitochondrial biogenesis and thermogenic differentiation. The upregulation of PPAR signaling pathway genes confirms the essential function of PPAR nuclear receptors downstream of cPGI_2_ (8). The top 10 most significantly enriched pathways from 159 KEGG pathways in the cPGI_2_-up-regulated gene fraction included oxidative phosphorylation, TCA cycle, fatty acid metabolism, and other metabolic pathways (Table 1). This result demonstrates that differentiation toward an oxidative adipocyte phenotype is the main and specific response of progenitors to prolonged cPGI_2_ treatment.

**Table 1:** **Top 10 most significantly enriched gene sets in the cPGI_2_-up-regulated gene fraction following 8 days of progenitor differentiation**.

Gene set name^a^	Size	Enrichment score^b^	FDR *q*-value
KEGG_PARKINSONS_DISEASE^c^	87	0.77	<0.0001
KEGG_OXIDATIVE_PHOSPHORYLATION	89	0.77	<0.0001
KEGG_HUNTINGTONS_DISEASE^c^	129	0.67	<0.0001
KEGG_PEROXISOME	57	0.74	<0.0001
KEGG_ALZHEIMERS_DISEASE^c^	121	0.64	<0.0001
KEGG_CITRATE_CYCLE_TCA_CYCLE	25	0.82	<0.0001
KEGG_FATTY_ACID_METABOLISM	35	0.72	0.0001
KEGG_PPAR_SIGNALING_PATHWAY	54	0.67	0.0001
KEGG_VALINE_LEUCINE_AND_ISOLEUCINE_DEGRADATION	40	0.65	0.001
KEGG_FRUCTOSE_AND_MANNOSE_METABOLISM	22	0.72	0.0026

*^a^Lin^−^CD29^+^CD34^+^Sca-1^+^ cells were cultured in adipogenic media ± cPGI_2_ for 8 days. RNA expression profiling was performed with Illumina beadchip arrays (n = 3). Global expression profiles (cPGI_2_ vs. Control) were subjected to gene set enrichment analysis (GSEA) with the KEGG pathway gene set collection. Gene sets were ranked by the False Discovery Rate (FDR) q-value reflecting significance*.

*^b^Maximum of the corresponding enrichment score curve*.

*^c^The indicated gene sets are overlapping >50% with the KEGG_OXIDATIVE_PHOSPHORYLATION gene set*.

The question remained as to the extent to which the cPGI_2_-induced cell phenotype resembles a classical brown adipocyte phenotype. To this end, we performed principal component analysis to compare global gene expression in cPGI_2_-treated and control cells at day 8 as well as adipocytes from classical BAT-derived progenitors. As a reference we included undifferentiated progenitors from posterior subcutaneous fat (White d0). The three types of mature adipocytes aligned with each other and separately from undifferentiated cells on principal component 1 (PC1), likely reflecting similar degrees of adipogenesis (Figure 1E). PC2 mainly represents the differences between the adipocyte types and reveals an intermediate phenotype of cPGI_2_-treated cells between the white and brown cell phenotypes. To further delineate this, we examined the expression pattern of cPGI_2_-regulated genes in the comparison of classical brown vs. control white adipocytes (without cPGI_2_ treatment). One thousand seven hundred ninety-three of 3589 cPGI_2_-regulated genes (cPGI_2_ vs. Control, *p* < 0.05 at day 8) were also differentially expressed in brown vs. white/control adipocytes (*p* < 0.05). Remarkably, the expression patterns were highly concordant in the two comparisons (Figure 1F), and this was not due to varying differentiation efficiencies compared to white control cultures (Figure S3A in Supplementary Material). In addition, there was no concordance of cPGI_2_-dependent expression with the general adipogenic differentiation program (day 8 vs. 0, Figure S3B in Supplementary Material). Taken together, these results suggest that cPGI_2_ broadly promotes a brown-like gene expression program independently of any effects on adipogenic differentiation.

To test whether cPGI_2_ influences developmental lineage commitment, we analyzed the expression of genes known to be brown lineage-selective, but could not detect a consistent cPGI_2_ effect on *Ebf3*, *Lhx8*, or *Zic1* (Figure 1G) (21, 22). In addition, cPGI_2_ did not consistently alter the expression of beige-selective genes including *Tnfrsf9* (CD137), *Tbx1*, *Hoxc9*, and *Slc36a2* (Pat2) (22–24). Taken together, our findings suggest that cPGI_2_ promotes oxidative brown-like differentiation without inducing a major lineage switch or enrichment.

### Synergism of cPGI_2_ signaling during progenitor activation and beige/brite adipocyte maturation results in the late activation of *Ucp1* transcription

**Figure 2:**
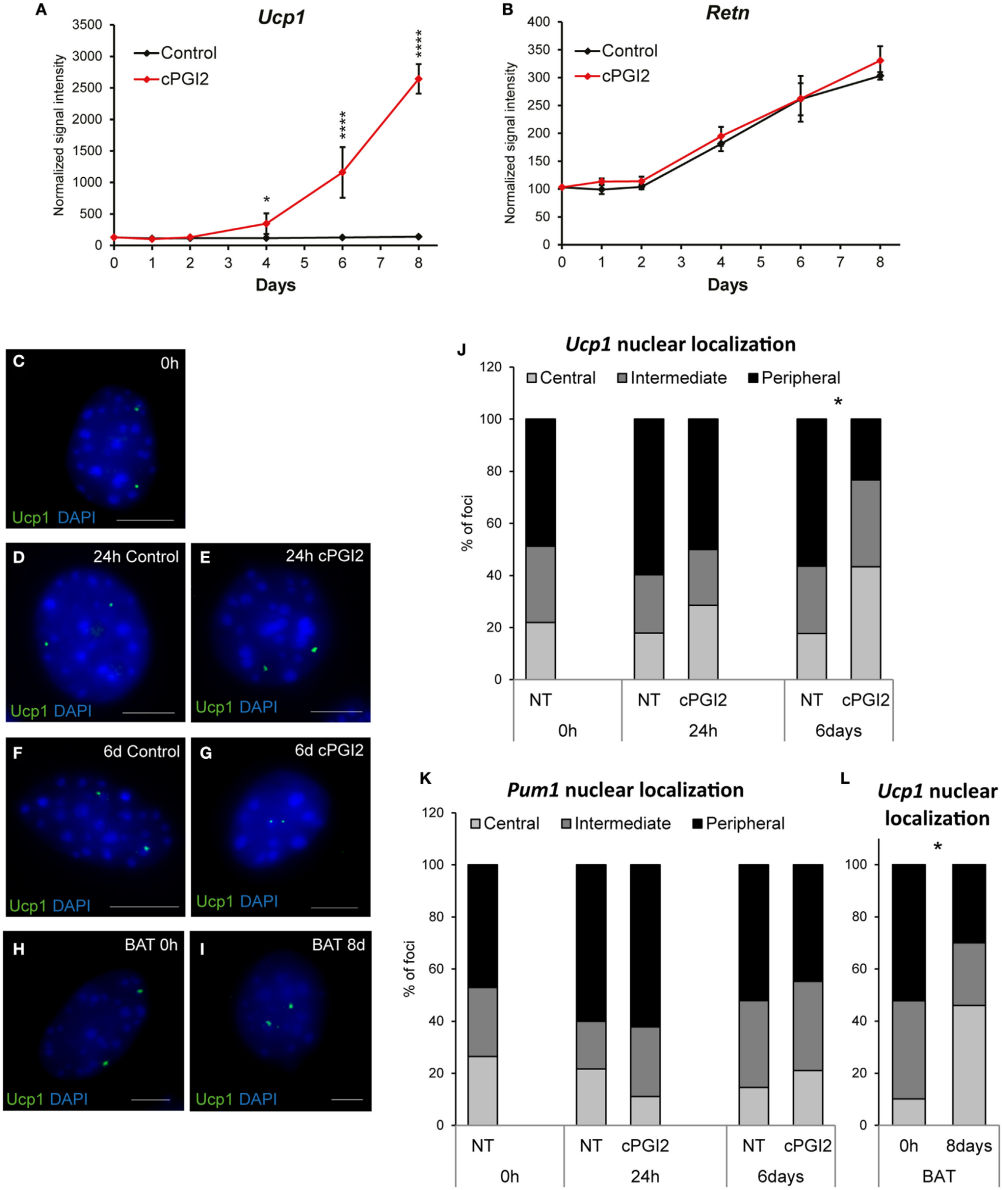
**Late non-linear upregulation of *Ucp1* expression by cPGI_2_ and associated late nuclear relocalization of the *Ucp1* gene locus**. Lin^−^CD29^+^CD34^+^Sca-1^+^ cells were cultured in adipogenic media ± cPGI_2_. **(A,B)** RNA was obtained at the indicated time points for expression profiling with Illumina beadchip arrays (*n* = 3). Normalized signal intensities for the indicated genes/probes are shown (asterisks indicate Bonferroni cPGI_2_ vs. Control *p < 0.05, ****p < 0.0001). **(C–L)** Cells were fixed for 3D nuclear architecture-preserving DNA–FISH analysis in the undifferentiated state (0 h) **(C)** and at 24 h **(D,E)** or 6 days **(F,G)** of differentiation with **(E,G)** or without **(D,F)** cPGI_2_. Cells from interscapular BAT (without cPGI_2_) were analyzed at 0 h or 8 days **(H,I)**. The *Ucp1* gene locus **(C–I)** was detected using an AlexaFluor^®^ 488-5-dUTP-labeled probe (green) and the cells were stained with DAPI (blue). Representative images of progenitor and adipocyte nuclei are shown (scale bar 10 μm). **(J)** For quantitative analysis of *Ucp1* localization, the nuclei were segmented into three shells (Central/Intermediate/Peripheral). The distribution of *Ucp1* loci under the indicated conditions is shown (* indicates χ^2^ test cPGI_2_ vs. Control *p* = 2.4 × 10^−8^, *n* = 30–50). **(K)** Distribution of the *Pum1* locus as detected in Figures S4B,C in Supplementary Material. **(L)** Distribution of the *Ucp1* locus in cells from interscapular BAT (* indicates χ^2^ test 8 days vs. 0 h *p* = 4.8 × 10^−16^, *n* = 30–50).

Based on our results so far, it was not clear at which differentiation stage cPGI_2_ triggers progenitor browning. To address this, we first examined the time course of induction of brown marker genes in relation to the progress of adipogenesis. Whereas cPGI_2_ did not influence the linear upregulation of the adipogenic marker resistin (*Retn*), the induction of *Ucp1* by cPGI_2_ began between day 2 and 4 of treatment, but displayed exponential kinetics indicating the involvement of synergistic cPGI_2_ effects (Figures 2A,B).

The relocalization of gene loci away from the nuclear lamina and toward the center of the nucleus has been shown to be associated with their transcriptional activation during differentiation in mammalian cells (25–27). To gain more insight into the mode and kinetics of transcriptional regulation of thermogenic genes by cPGI_2_, we asked whether nuclear localization of *Ucp1* correlates with the induction of *Ucp1* transcription by cPGI_2_. Nuclear structure-preserving 3D-FISH revealed that in the undifferentiated state (0 h) the two *Ucp1* alleles had peripheral or intermediate localization in the majority of progenitor nuclei (Figures 2C,J). Induction of differentiation and cPGI_2_ treatment did not acutely alter this localization pattern (24 h, Figures 2D,E,J). Within the subsequent 5 days of cPGI_2_ treatment, the number and density of DAPI^bright^ chromocenters decreased, and remarkably, *Ucp1* loci significantly shifted toward the interior of the nuclei (Figures 2F,G,J), correlating with the late transcriptional activation by cPGI_2_ (Figure 2A). To control the specificity of this finding, we examined the nuclear localization of the Pumilio 1 (*Pum1*) gene locus, the expression of which was not affected by differentiation or cPGI_2_ treatment (Figure S4A in Supplementary Material). In accordance with the expression pattern, the nuclear localization of the *Pum1* locus was not altered by cPGI_2_ (Figure 2K; Figures S4B,C in Supplementary Material). In addition, we tested the nuclear localization of the *Ucp1* locus in adipocytes derived from interscapular brown fat progenitor cells, which express high levels of *Ucp1* mRNA without cPGI_2_ stimulation. Whereas undifferentiated BAT progenitors displayed mainly intermediate/peripheral nuclear localization of *Ucp1*, mature brown adipocytes had 46% central localization, which is comparable to 43.3% in cPGI_2_-treated adipocytes from subcutaneous white fat (Figures 2H,I,L). Taken together, these results are in line with a late transcriptional activation of *Ucp1* by cPGI_2_.

**Figure 3:**
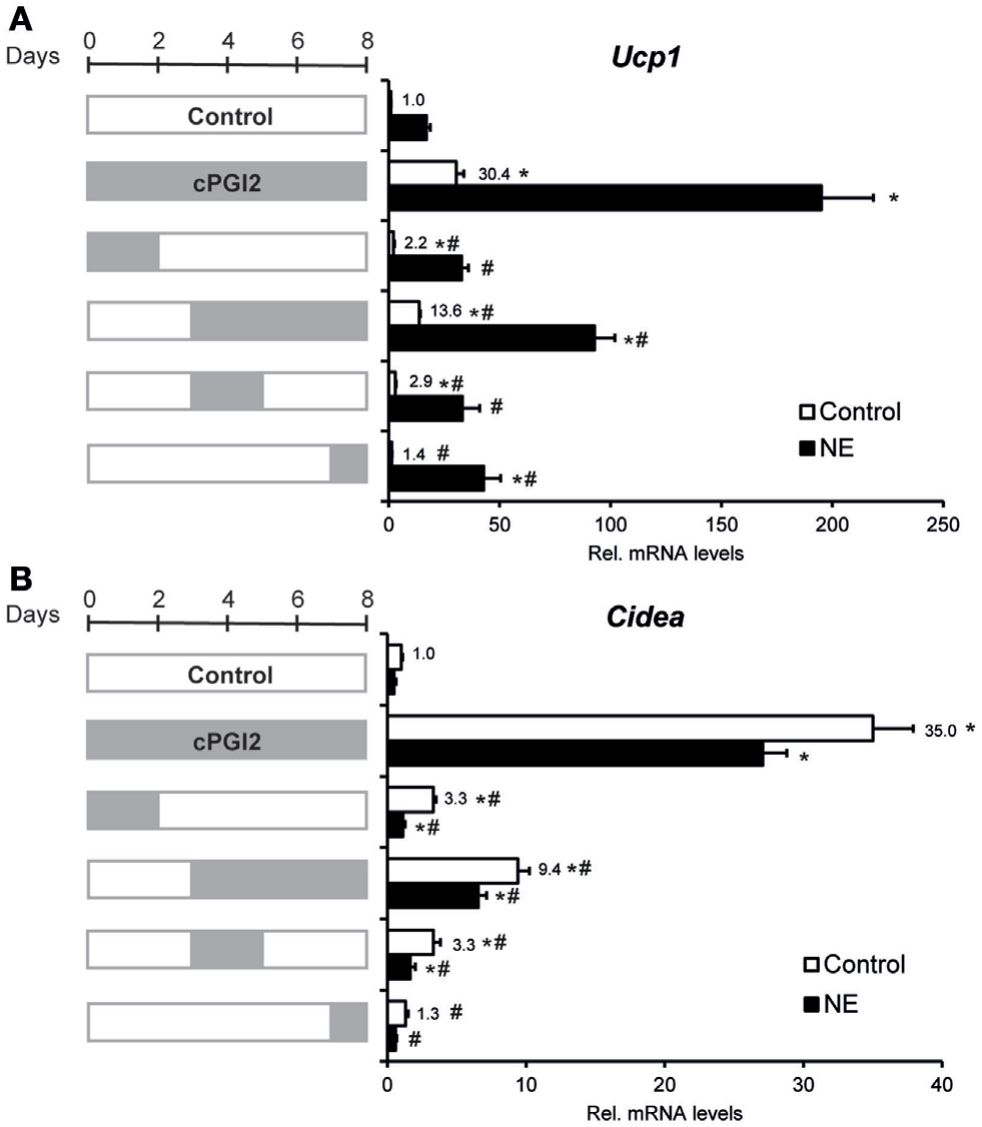
**Synergism of cPGI_2_ signaling during progenitor activation and adipocyte maturation for the induction of thermogenic marker genes**. Lin^−^CD29^+^CD34^+^Sca-1^+^ cells were cultured in adipogenic media for 8 days. cPGI_2_ was included in the media as indicated by the gray bars. Three hours before harvest, cells were cultured ± NE. RNA expression of *Ucp1* **(A)** and *Cidea* **(B)** was analyzed by qRT-PCR [* and ^#^ indicate Tukey *p* < 0.05 for the respective group vs. Control (0–8 days) and vs. cPGI_2_ (0–8 days), respectively, *n* = 3].

Given the late upregulation of *Ucp1* expression by cPGI_2_, we sought to define the role of late vs. early cPGI_2_ signaling. To define the differentiation time window in which cPGI_2_ stimulation promotes beige/brite differentiation, we restricted the duration of cPGI_2_ treatment (Figures 3A,B). Neither the early nor the late cPGI_2_ stimulation was sufficient to induce the full activation of *Ucp1* and *Cidea* expression. Intriguingly, early cPGI_2_ signaling during progenitor commitment (day 0–2) synergized with late cPGI_2_ stimulation during maturation (day 3–8) in a non-additive manner, highlighting an important role during progenitor activation.

### cPGI_2_ induces progenitor activation through cell cycle and adhesion pathways prior to metabolic maturation

**Figure 4:**
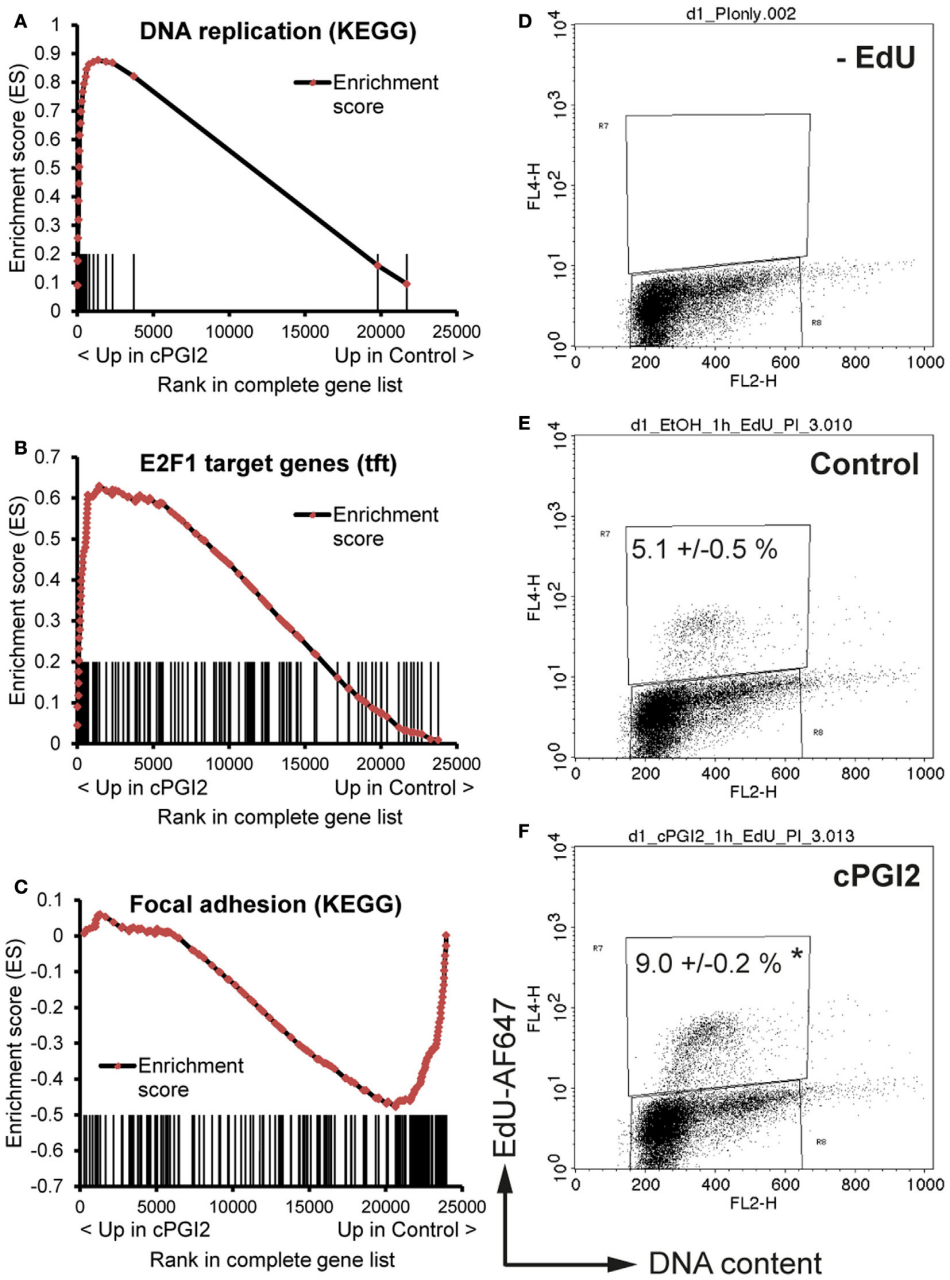
**cPGI_2_ induces progenitor activation through cell cycle and adhesion pathways**. Lin^−^CD29^+^CD34^+^Sca-1^+^ cells were cultured in adipogenic media ± cPGI_2_ for 24 h. **(A–C)** RNA was obtained for expression profiling with Illumina beadchip arrays (*n* = 3). Enrichment plots of the indicated gene sets were obtained by GSEA (cPGI_2_ vs. Control) with the KEGG pathway [**(A,C)**, see Table 2] or the transcription factor motif **(B)** gene set collections. Vertical bars represent the individual genes of the gene set/pathway ranked according to their regulation by cPGI_2_. *X*-axis values represent the rank within the complete ranked gene list (transcriptome). The enrichment score (ES) reflects the degree to which a gene set is overrepresented at the top or bottom of the complete ranked gene list. **(E,F)** Cells were pulse-labeled with EdU for 1 h, processed for EdU-AlexaFluor647 and propidium iodide staining, and analyzed by flow cytometry (*n* = 3). Cells cultured without EdU served as negative control **(D)**. The mean percentage of EdU^+^ cells is shown (* indicates *t*-test cPGI_2_ vs. Control *p* = 0.003).

We next sought to determine the transcriptional pathways underlying the early progenitor response to cPGI_2_. GSEA at 24 h of cPGI_2_ treatment using the KEGG pathway collection revealed the marked enrichment of cell cycle and proliferation pathways in the gene fraction up-regulated by cPGI_2_ (Table 2; Figure 4A).

**Table 2:** **Top 10 most significantly enriched gene sets in the cPGI_2_-up and down-regulated gene fraction at 24 h of progenitor differentiation**.

Gene set name^a^	Size	Enrichment score	FDR *q*-value
**ENRICHED IN THE cPGI_2_-UP-REGULATED GENE FRACTION**			
KEGG_DNA_REPLICATION	24	0.88	<0.0001
KEGG_HOMOLOGOUS_RECOMBINATION	22	0.86	<0.0001
KEGG_CELL_CYCLE	98	0.61	<0.0001
KEGG_SPLICEOSOME	71	0.65	<0.0001
KEGG_NUCLEOTIDE_EXCISION_REPAIR	31	0.73	<0.0001
KEGG_MISMATCH_REPAIR	15	0.85	<0.0001
KEGG_PYRIMIDINE_METABOLISM	73	0.6	0.0001
KEGG_BASE_EXCISION_REPAIR	25	0.72	0.0002
KEGG_NON_HOMOLOGOUS_END_JOINING	12	0.74	0.0196
KEGG_AUTOIMMUNE_THYROID_DISEASE	22	0.61	0.0276
**ENRICHED IN THE cPGI_2_-DOWNREGULATED GENE FRACTION**			
KEGG_FOCAL_ADHESION	157	−0.48	0.0032
KEGG_VASCULAR_SMOOTH_MUSCLE_CONTRACTION	89	−0.51	0.0054
KEGG_TIGHT_JUNCTION	88	−0.52	0.0043
KEGG_AXON_GUIDANCE	105	−0.46	0.0247
KEGG_REGULATION_OF_ACTIN_CYTOSKELETON	164	−0.43	0.0321
KEGG_LONG_TERM_DEPRESSION	58	−0.51	0.0288
KEGG_LEUKOCYTE_TRANSENDOTHELIAL_MIGRATION	85	−0.46	0.0284
KEGG_ADHERENS_JUNCTION	58	−0.51	0.0327
KEGG_ENDOCYTOSIS	117	−0.44	0.0371
KEGG_ECM_RECEPTOR_INTERACTION	69	−0.47	0.0386

*^a^Lin^−^CD29^+^CD34^+^Sca-1^+^ cells were cultured in adipogenic media ± cPGI_2_ for 24 h. RNA expression profiling was performed with Illumina beadchip arrays (n = 3). Global expression profiles (cPGI_2_ vs. Control) were subjected to GSEA with the KEGG pathway gene set collection. Gene sets were ranked by the False Discovery Rate (FDR) q-value reflecting significance*.

Furthermore, we applied GSEA with gene sets consisting of genes which share specific conserved transcription factor motifs in their promoter regions (15). In this way, we found that genes with E2F1 motifs were highly up-regulated by cPGI_2_ at 24 h (Figure 4B). The transcription factor E2F1 is known to promote G1–S cell cycle transition and S phase progression (28), and thus, cPGI_2_ may be inducing S phase gene expression, including DNA replication genes, through modulation of E2F1 activity. To test whether these cPGI_2_-mediated gene expression changes resulted in transient progenitor proliferation at the onset of differentiation, we pulse-labeled cells at 24 h of cPGI_2_ treatment with the nucleotide analog EdU and measured incorporation into genomic DNA by flow cytometry (Figures 4D–F). Indeed, cPGI_2_ caused a significant increase in the proportion of EdU^+^ cycling cells (from 5.1 to 9% within 1 h of labeling). Interestingly, the top 10 gene sets from the KEGG pathway collection (GSEA) in the cPGI_2_-downregulated gene fraction at 24 h were dominated by cell adhesion and cytoskeletal pathways (Table 2; Figure 4C), indicating that cPGI_2_ promotes changes in cell adhesion and morphology associated with progenitor activation in parallel to cell cycle effects.

**Figure 5:**
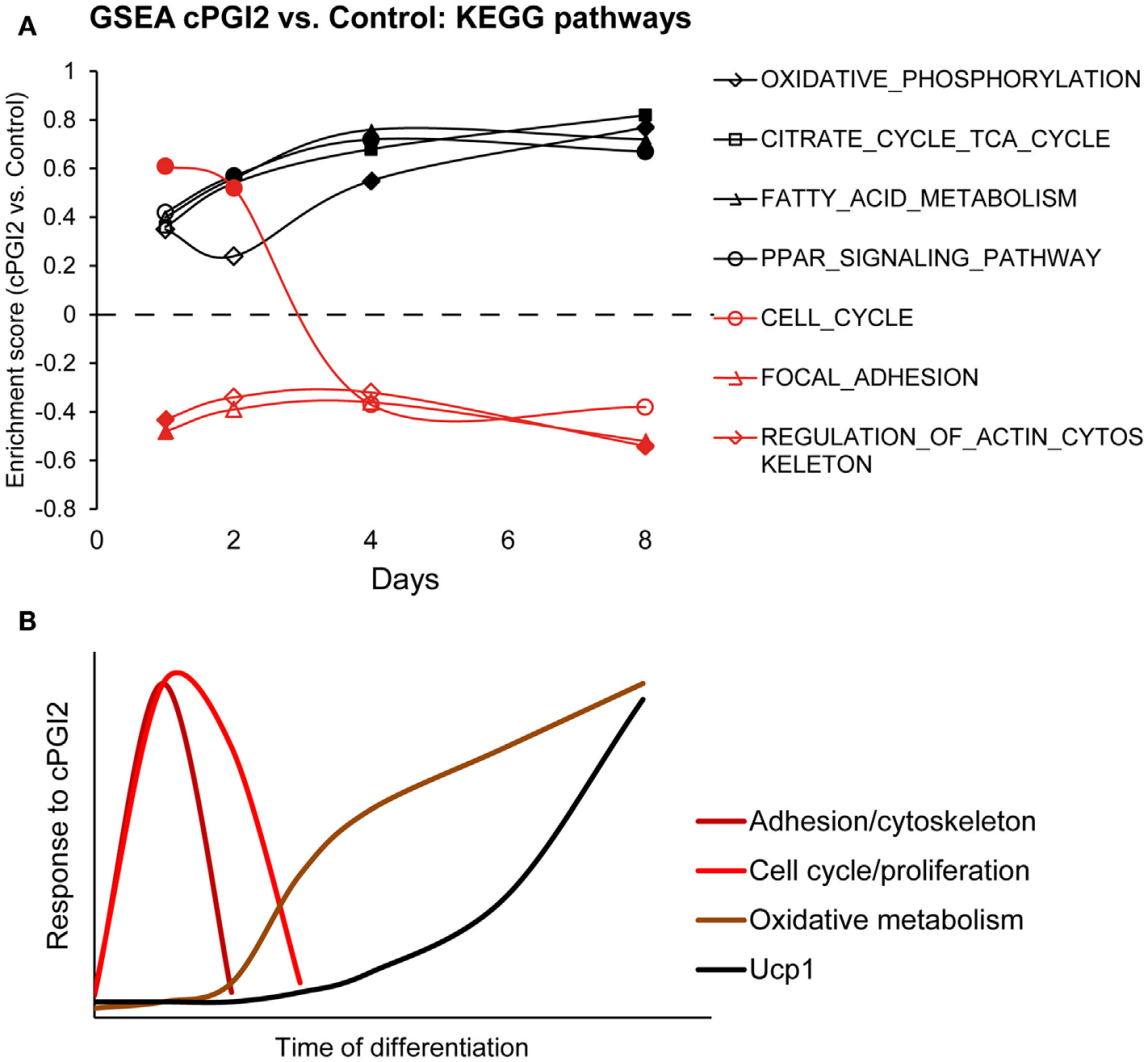
**Progenitor activation by cPGI_2_ precedes metabolic differentiation**. **(A)** Lin^−^CD29^+^CD34^+^Sca-1^+^ cells were cultured in adipogenic media ± cPGI_2_ for the indicated times, at which RNA was obtained for expression profiling with Illumina beadchip arrays (*n* = 3). GSEA was performed at each time point (cPGI_2_ vs. Control) with the KEGG pathway gene set collection. The enrichment scores of the indicated gene sets related to progenitor activation (red) or metabolic maturation (black) are plotted. Closed markers indicate significance (FDR *q* < 0.05). **(B)** Schematic summary of the kinetics of progenitor cell responses to cPGI_2_ as detected by gene expression assays, GSEA and EdU incorporation analysis.

In order to determine the temporal order of progenitor responses to cPGI_2_ and their relation to the metabolic differentiation toward the oxidative thermogenic phenotype, we performed GSEA across the time course of cPGI_2_ treatment, i.e., cPGI_2_ vs. Control at each time point (Figure 5A). Intriguingly, the cytoskeletal and adhesion changes as well as the transient cell cycle activation by cPGI_2_ preceded the upregulation of oxidative pathways including Ppar target genes. Taken together, our findings suggest that the synergism between early and late cPGI_2_ signaling results from the early activation of progenitors and priming for the later induction of oxidative/thermogenic genes by cPGI_2_ (Figure 5B).

### A simplified method for the prospective isolation of defined progenitors for beige/brite differentiation

**Figure 6:**
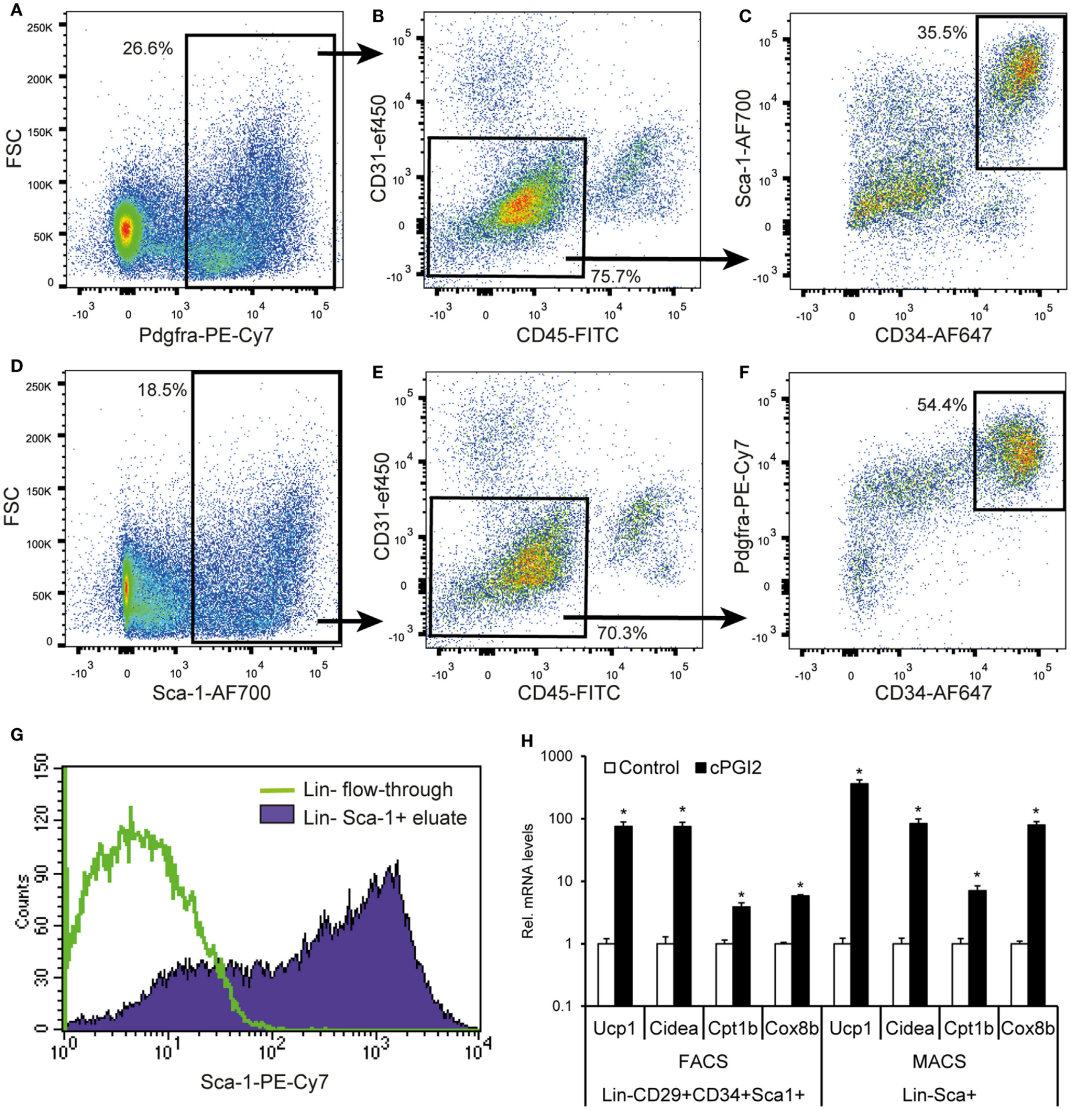
**A MACS selection procedure for the prospective isolation of progenitor cells for beige/brite differentiation**. **(A–F)** Single cell suspensions were obtained by collagenase digestion of subcutaneous fat, and stained for six-color FACS with the indicated antibodies after removal of adipocytes by centrifugation and erythrocytes by TER119-MACS^®^ depletion. Debris and singlets were excluded and selected through FSC/SSC and FSC-A/H, respectively. **(A–C)** and **(D–F)** represent independent gating schemes. Values (%) indicate cells % of parent plot. Representative plots from multiple independent experiments are shown. **(G)** Erythrocytes, leukocytes, and endothelial cells were removed from single cell suspensions in a first MACS step with biotinylated Ter119, CD45, and CD31 antibodies and streptavidin-conjugated microbeads. Sca-1^+^ cells were enriched in a second MACS step with Sca-1-PE-Cy7 antibody and anti-PE-Cy7 microbeads. The resulting cell population (Lin^−^Sca-1^+^ eluate) as well as the Lin^−^ flow-through were subjected to flow cytometry. Comparable purities were obtained with directly conjugated antibody-bead combinations (data not shown). **(H)** MACS- and FACS-purified cells were cultured and differentiated for 8 days in the presence or absence of cPGI_2_ (see Materials and Methods) before subjected to RNA expression analysis by qRT-PCR (* indicates *t*-test *p* < 0.003 cPGI_2_ vs. Control, *n* = 3).

Finally, we aimed at developing a more accessible method for the isolation of progenitors with beige/brite potential based on magnetic bead separation with few specific markers. Since Pdgfra is the only currently known marker of beige/brite progenitors proven by genetic lineage tracing, we assessed its potential as a single marker for the prospective isolation of beige/brite progenitors. As reported by others, Pdgfra expression was detectable in a fraction of Lin^+^ cells, in particular, CD45^+^ leukocytes (Figures 6A,B) (19). Nevertheless, 76% of Pdgfra^+^ cells were Lin^−^. Importantly, though, only 36% of Lin^−^Pdgfra^+^ cells were CD34^+^Sca-1^+^ and thereby adipogenic (Figure 6C). This expression pattern suggests that Pdgfra-based cell isolation would not result in highly specific enrichment of adipogenic/beige/brite progenitors.

We went on to assess the suitability of Sca-1 as a marker for the enrichment of adipogenic cells includes beige/brite progenitors, given that the majority of Lin^−^CD29^+^CD34^+^Sca1^+^ cells were Pdgfra^+^ (Figure S1 in Supplementary Material). Seventy percent of Sca-1^+^ cells were Lin^−^, and the Lin^−^Sca-1^+^ population contained mainly Pdgfra^+^ cells, which were CD34^+^ to a high proportion (54% of the Lin^−^Sca-1^+^ population) (Figures 6D–F).

On this basis, we went on to develop a two-step magnetic separation procedure with a negative selection of Lin^+^ cells (erythroid Ter119^+^, CD31^+^ and CD45^+^) followed by positive selection of ­Sca-1^+^ cells. This approach led to quantitative recovery of Sca-1^+^ cells with a purity of approximately 75% (Figure 6G). To confirm that the Lin^−^Sca-1^+^ MACS-purified cells retain the beige/brite adipogenic potential compared to the Lin^−^CD29^+^CD34^+^Sca1^+^ population, we cultured both cell isolates under adipogenic conditions in the presence of cPGI_2_. We examined the expression of *Ucp1*, *Cidea*, *Cpt1b*, and *Cox8b*, known thermogenic adipocyte markers ranking within the 10 most significantly regulated genes in the expression profiles (day 8 cPGI_2_ vs. Control, adjusted *p* < 10^−6^). The expression of all markers increased markedly and to a similar extent by cPGI_2_ in both cell types (Figure 6H). Taken together, we demonstrate the MACS-separated Lin^−^Sca-1^+^ cells represent a good approximation of the Lin^−^CD29^+^CD34^+^Sca1^+^Pdgfra^+^ population retaining the capacity for the induction of beige/brite differentiation.

## Discussion

An increasing number of molecular factors have been established as regulators of adipose tissue browning through the use of genetic mouse models (1). However, the investigation of the signaling and transcriptional pathways downstream of physiological mediators of progenitor-dependent beige/brite differentiation has been hampered, partly by the paucity of appropriate cell models. Our study contributes to this challenge in two ways. (a) We present a cell model of beige/brite differentiation based on defined primary adipose tissue progenitor cells and the physiological inducer PGI_2_. (b) Using time course expression profiling, we dissect the cascade of progenitor cell responses during beige/brite differentiation, and show that early progenitor activation by cPGI_2_ through cell cycle and morphology pathways precedes and synergizes with the late upregulation of thermogenic gene expression.

Although certain cell surface markers have been shown to be preferentially expressed in beige progenitor cells and used for their enrichment, we focused on markers based on previous lineage tracing evidence (7, 19, 22). So far, Pdgfra has been the only marker shown by lineage tracing to be expressed in beige/brite progenitors. However, we and others could show that expression of Pdgfra is not restricted to adipogenic cells (Figures 6A–C) (19). The Lin^−^CD29^+^CD34^+^Sca-1^+^ population, which was shown to contain the majority of adipogenic cells, homogeneously expressed Pdgfra (Figures S1A–C in Supplementary Material) (18, 19). On this basis, we used this marker combination for FACS-isolation, and furthermore, developed a simplified magnetic enrichment procedure yielding cells with beige/brite potential comparable to FACS-isolated cells (Figure 6). Of note, the simplified isolation method could also be applicable for addressing questions related to general (“white”) adipogenesis from defined progenitors, if cPGI_2_ treatment is omitted.

Wu et al. provided evidence for the existence of distinct committed progenitors for white vs. beige/brite adipocytes (22). Alternatively, bipotential white/beige progenitors have been implicated in browning (7). Our cell model for the investigation of beige/brite adipocyte recruitment from progenitors is applicable to both hypotheses, as it is likely to include all adipogenic cells including beige/brite progenitors. Notably, our data do not indicate enrichment of beige/brite lineage cells or a lineage switch by cPGI_2_ (Figure 1G).

An emerging concept on the induction of progenitor responses and adipose tissue browning suggests a key role of transient inflammatory signals at the onset of the remodeling process (29–31). In this light, the function of COX-2 and prostaglandins in the recruitment of beige/brite adipocytes becomes evident. In addition, the suitability of cPGI_2_ as a physiological inducer of beige/brite differentiation is supported by the following facts. (i) cPGI_2_ caused broad and specific induction of the thermogenic expression program without strong effects on general adipogenesis (Figure 1; Figures S2 and S3 in Supplementary Material; Table 1). (ii) It is able to induce thermogenic marker genes in primary human adipose tissue progenitors (8) as well as in the human hMADS model (Ez-Amri, personal communication). (iii) cPGI_2_ relays NE signaling to the progenitor level and activates Ptgir-cAMP as well as Ppar pathways, which are central to thermogenic differentiation (2, 8, 12, 13).

The induction of beige/brite differentiation by the Pparg agonist rosiglitazone has been used for the initial definition of brite adipogenesis (32). However, this inducer causes supra-physiological activation of Pparg and potently promotes general adipogenesis, which is the reason for its broad usage in adipogenic differentiation media (33, 34). An alternative robust model for beige/brite differentiation involves treatment of progenitors from white fat with bone morphogenetic protein 7 (Bmp7), even though the function of Bmp7 in browning *in vivo* remains to be solidified (35).

Time course profiling of the progenitor response to cPGI_2_ revealed that the upregulation of thermogenic genes occurred late in the differentiation process (Figures 2 and 5). The late induction was accompanied by a profound reorganization of the nuclear architecture and relocalization of the *Ucp1* gene locus from the nuclear periphery to central territories, which to our knowledge has not been reported previously. This relocalization was also observed during the differentiation of adipocyte progenitors from brown fat, implying a general functional relevance. Thus, our cell model could serve the exploration of this novel potential level of regulation of *Ucp1* expression.

Despite the late upregulation of thermogenic markers, we could show that the full induction of these genes required the synergistic action of early and late cPGI_2_ signaling. Focusing on the early cPGI_2_-mediated transcriptional pathways, we detected changes indicative of transient cPGI_2_-induced cell cycling at 24 h, which was confirmed by EdU incorporation analysis (Figures 4 and 5; Table 2). Whereas mitotic clonal expansion has been shown to play a role in adipogenesis, a link to beige/brite differentiation has not been reported (36). Importantly, increased cell cycling was not associated with increased adipogenesis in cPGI_2_-treated cells (Figures 1 and 2; Figures S2 and S3 in Supplementary Material). The mechanistic link between progenitor cycling and the commitment to beige/brite differentiation is currently under investigation. A connecting node could be the retinoblastoma protein, the inactivation of which has been shown to promote G1–S progression as well as thermogenic adipocyte differentiation (37, 38). In addition to cell cycling, cPGI_2_ affected additional pathways related to progenitor activation, namely, the early downregulation of cell adhesion and cytoskeletal pathways (Figures 4 and 5; Table 2). Recently, the MRTF/SRF transcription factors were implicated in the regulation of beige/brite differentiation downstream of cytoskeletal changes (39). It is tempting to speculate that cPGI_2_-mediated morphological responses are causally related to the priming of thermogenic gene expression. Overall, our results highlight the importance of core progenitor activation and commitment pathways for the recruitment of thermogenic cells.

According to current theory, beige/brite thermogenic adipocytes can be recruited from immature progenitors as well as from mature cells (2). Independently of the degree of contribution of each path to physiological browning in rodents, the proliferation capacity and plasticity of progenitor cells highlight their potential for the therapeutic recruitment of thermogenic cells in the context of metabolic disease. Understanding the biology of primary adipocyte progenitor cells is a prerequisite in this direction.

## Conflict of Interest Statement

Stefan Wild, Andreas Bosio, and Olaf Hardt are full time employees of Miltenyi Biotec GmbH. The remaining co-authors declare that the research was conducted in the absence of any commercial or financial relationships that could be construed as a potential conflict of interest.

## Supplementary Material

The Supplementary Material for this article can be found online at http://journal.frontiersin.org/article/10.3389/fendo.2015.00129

## Footnotes

^1^www.ebi.ac.uk/arrayexpress

^2^http://www.broadinstitute.org/gsea/msigdb/index.jsp

^3^http://www.gene-regulation.com/
