# Supplementary material for: Transcriptional Pathways in cPGI2-Induced Adipocyte Progenitor Activation for Browning
Source: Front Endocrinol (Lausanne). 2015 Aug 17;6:129. doi: 10.3389/fendo.2015.00129 (PMC4538297; doi:10.3389/fendo.2015.00129)
Supplement: Supplementary file 1 [file Presentation_1.PDF]

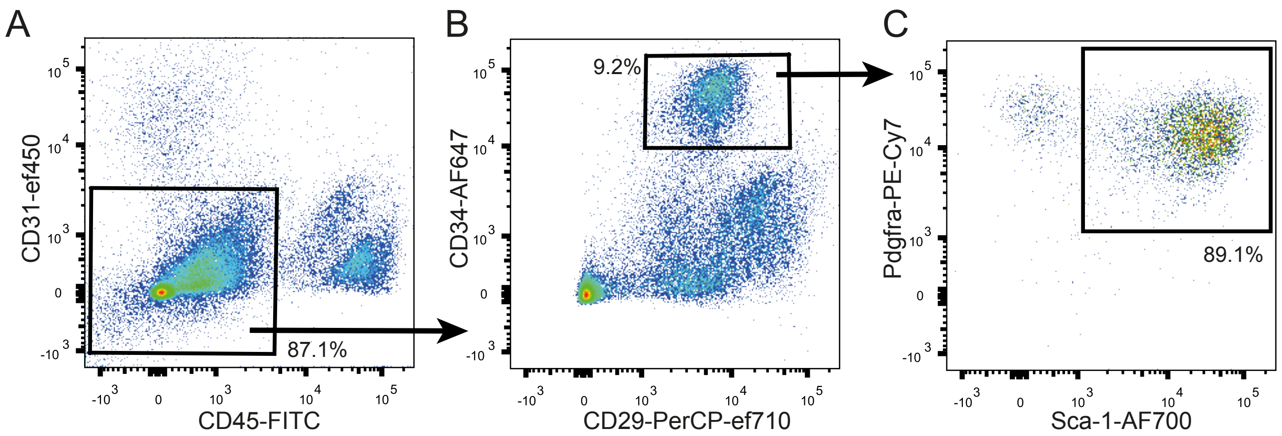

Figure S1. Definition of Pdgfra$^+$ adipose tissue progenitor cells by 6-color FACS for prospective isolation and beige/brite differentiation. Single cell suspensions were obtained by collagenase digestion of subcutaneous fat, and stained with the indicated antibodies after removal of adipocytes by centrifugation and erythrocytes by TER119-MACS® depletion. Debris and singlets were excluded and selected through FSC/SSC and FSC-A/H respectively. Panels A-C represent the gating scheme. Values (%) indicate cells % of parent plot. Representative plots from multiple independent experiments are shown.

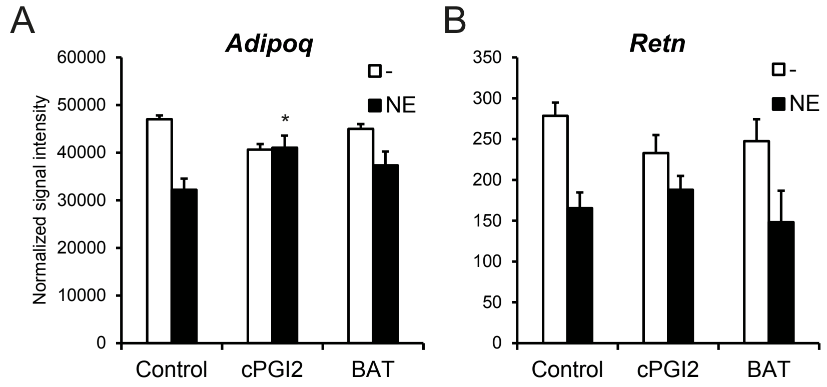

Figure S2. Expression of general adipogenic marker genes in $cPGI_2$-treated cells. $Lin^-CD29^+CD34^+Sca\text{-}1^+$ cells from posterior subcutaneous fat were cultured in adipogenic media +/- $cPGI_2$ for 8 days. $Lin^-CD29^+CD34^+Sca\text{-}1^+$ cells from interscapular brown fat (BAT) were cultured in adipogenic media for 8 days and used as a reference. 3 hours before harvest, cells were cultured +/- norepinephrine (NE). RNA expression profiling was performed with Illumina beadchip arrays (n=3). (A,B) Normalized signal intensities for the indicated genes/probes are shown (asterisks indicate Bonferroni $cPGI_2$ vs. Control *$p<0.05$, n=3).

A

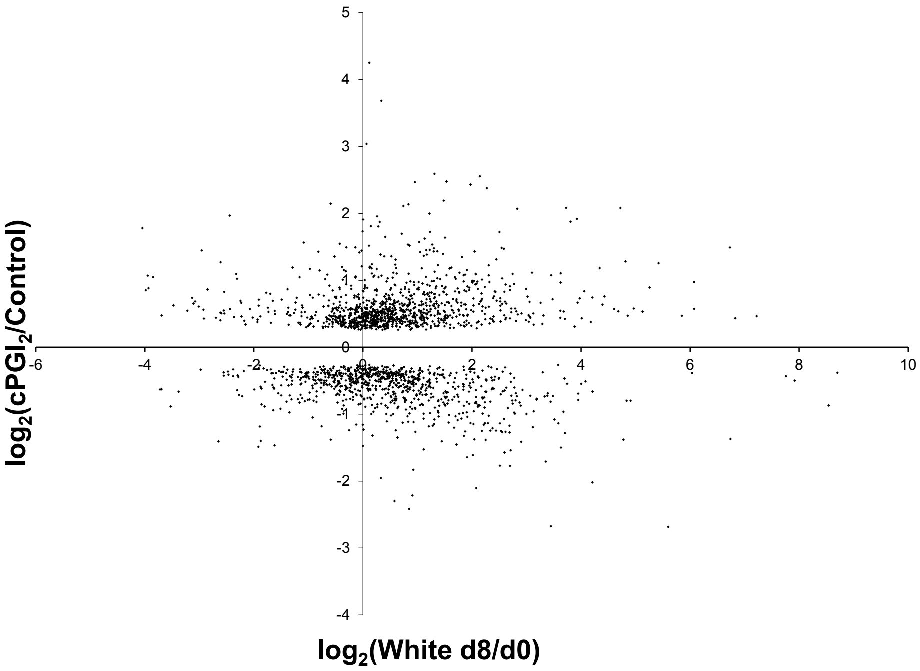

B

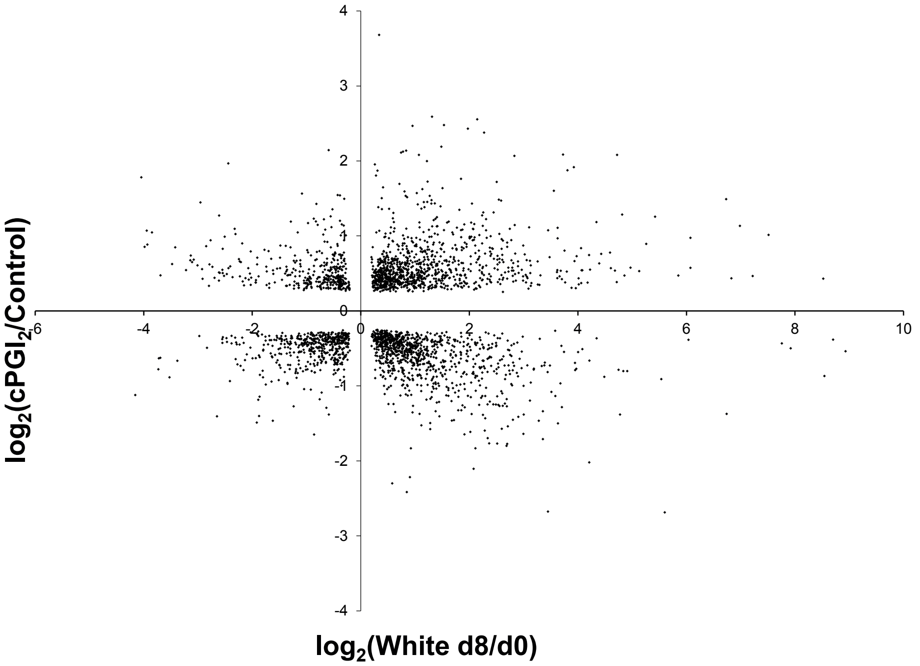

Figure S3. Lack of concordance of $cPGI_2$-mediated gene expression with the general adipogenesis expression program. $Lin^-CD29^+CD34^+Sca\text{-}1^+$ cells from posterior subcutaneous fat were cultured in adipogenic media +/- $cPGI_2$ for 8 days. In parallel, $Lin^-CD29^+CD34^+Sca\text{-}1^+$ cells from interscapular brown fat (BAT) were cultured in adipogenic media for 8 days. RNA expression profiling was performed with Illumina beadchip arrays including samples from undifferentiated $Lin^-CD29^+CD34^+Sca\text{-}1^+$ cells from posterior subcutaneous fat (White d0) (n=3). (A) 1793 genes were selected with significant differential expression ($p<0.05$) in both the $cPGI_2$ vs. Control and the BAT vs. Control ('white') comparisons (see Fig. 1F). The log2-ratios of the corresponding expression levels in the $cPGI_2$ vs. Control and the White d8 vs. d0 comparisons were plotted. (B) 2795 genes were selected with significant differential expression ($p<0.05$) in both the $cPGI_2$ vs. Control and the White d8 vs. d0 comparisons. The log2-ratios of the corresponding expression levels in the two comparisons were plotted.

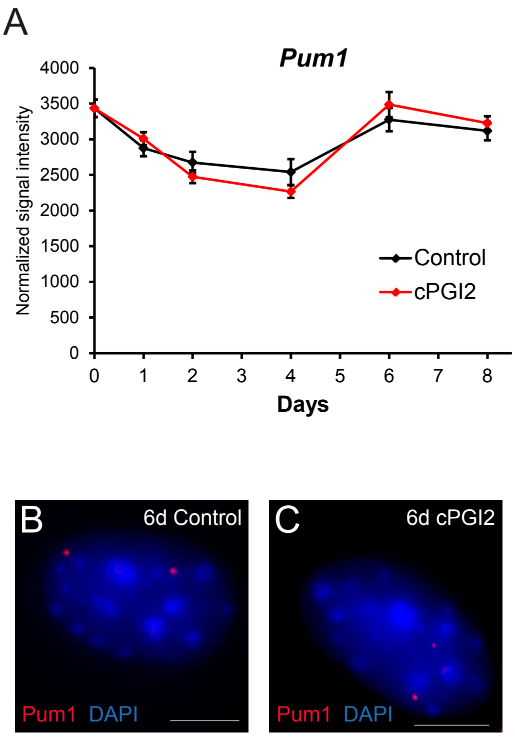

Figure S4. mRNA expression and nuclear localization of the *Pum1* gene during differentiation and $cPGI_2$ treatment. $Lin^-CD29^+CD34^+Sca\text{-}1^+$ cells from subcutaneous fat were cultured in adipogenic media +/- $cPGI_2$. (A) RNA was obtained at the indicated time points for expression profiling with Illumina beadchip arrays (n=3). Normalized signal intensities are shown. (B,C) Cells were fixed for 3D nuclear architecture-preserving DNA-FISH analysis in the undifferentiated state (0h) and at 24 h or 6 days (B,C) of differentiation with (C) or without (B) $cPGI_2$. The *Pum1* gene locus was detected using an Atto647N-dUTP-NT-labelled probe (red) and the cells were stained with DAPI (blue). Representative images of progenitor and adipocyte nuclei are shown for 6 days (scale bar 10µm).
